# Relationship between time elapsed since completion of radiotherapy and quality of life of patients with breast cancer

**DOI:** 10.1186/s12885-018-4207-y

**Published:** 2018-03-20

**Authors:** Jing-Jie Zhang, Hang Shu, Shuai-Shuai Hu, Yang Yu, Yi Sun, Yin Lv

**Affiliations:** 1 Department of Breast surgery, The First Affiliated Hospital of Anhui Medical University, Hefei, 230022 China; 2 Anhui Medical University, Hefei, 230000 China; 3 Department of Radiotherapy, The First Affiliated Hospital of Anhui Medical University, No. 281 Jixi Road, Hefei, Anhui 230022 China

**Keywords:** Quality of life, Breast cancer, Time after Radiotherapy, EORTC

## Abstract

**Background:**

To investigate the relationship between time elapsed since completion of radiotherapy (RT) and quality of life (QOL) of patients with breast cancer.

**Methods:**

A total of 300 patients with breast cancer were treated at the First Affiliated Hospital of Anhui Medical University between January 2013 and April 2016. Of these, 212 patients were included in the study. Patients were divided into 4 groups based on the time elapsed since completion of RT. The generic cancer questionnaire, EORTC QLQ-30, and the breast cancer-specific questionnaire, QLQ-BR23, were used to assess the QOL.

**Results:**

Analysis of time elapsed since completion of RT and QOL revealed changes in the scores for role function with passage of time; the third year’s scores were the highest. Pain symptoms during the 3rd and 4th years after RT were lower than those during the 1st and 2nd years after RT; scores for financial difficulties fluctuated with passage of time; perception of own body scores improved within first 3 years; sexual activity and enjoyment of sexual activity showed a significant decrease during the 2nd to 4th year post RT. Scores pertaining to concerns about future state of health showed a significant increase during the 2nd to 4th year after RT, while breast symptoms score showed fluctuations with passage of time.

**Conclusions:**

Social function, pain symptoms, and concerns about future state of health tended to improve with passage of time after RT. Other scales showed no correlation with time elapsed since completion of RT.

## Background

Globally, breast cancer is the most common malignancy and the leading cause of cancer-related deaths among women [1, 2]. In the United States, one in eight women will develop breast cancer during their lifetime [3]. Management of breast cancer includes surgery followed by radiotherapy (RT). Other treatment approaches include chemotherapy, endocrine therapy, and molecular targeted therapy [4]. Breast cancer remission in most cases is accompanied by concomitant short and/or long term damage to the physiological structure and function [5–10]. This could ultimately limit the patients’ activities of daily living and represent a source of long term impairment [8–14].

Advances in early diagnosis and treatment of breast cancer have helped improve patient survival. Consequently, the number of people living with disease sequelae is projected to increase over time. Providing long-term health care to these patients and measuring the impact of these services are pragmatic approaches. Assessment of cancer-specific quality of life (QOL) is an ideal approach towards this end. The European Organization Research and Treatment of Cancer (EORTC) QLQ-C30 questionnaire is a widely used instrument to assess the QOL of cancer patients [15]. Its specific module on breast cancer is the EORTC QLQ-BR23 [16]. Several studies have focused on the association between various treatment modalities (such as, surgical approach, radiation and chemotherapy regime or endocrine therapy) and QOL of patients with breast cancer [17]. However, few studies have investigated the temporal changes in QOL of these patients after completion of radiotherapy.

In the present study, we assessed the QOL of breast cancer patients at different time intervals after completion of RT using EORTC QLQ-C30 and EORTC QLQ-BR23 questionnaires. The objective was to assess whether time elapsed since completion of RT has a bearing on the QOL of these patients.

## Methods

### Study design

Patients with breast cancer were recruited at the Department of Radiation Oncology at the First Affiliated Hospital of Anhui Medical University, from January 2013 to April 2016. Breast cancer patients who received RT post surgery were enrolled in the study. Written informed consent was obtained from all patients prior to their enrolment. The study was approved by the ethics committee of The First Affiliated Hospital of Anhui Medical University.

### Implementation of questionnaire survey

Patients were divided into four groups based on time elapsed since completion of radiotherapy (0–1.5 years; 1.5–2.5 years; 2.5–3.5 years; and 3.5–4.5 years). Patients were administered both EORTC QLQ-C30 and QLQ-BR23 questionnaires. QLQ-C30 is a generic questionnaire used to assess patients with various tumors, while QLQ-BR23 is a breast cancer-specific questionnaire.

### Statistical analysis

Epidata 3.0 was used for data entry. Data are presented as mean ± standard deviation (SD) or as frequencies (percentages). ANOVA test was used to compare the QOL at different time intervals post RT. Multiple regression model was used to conduct multivariate analysis. Variables included in the model were age, educational level, surgical technique, surgical staging, number of chemotherapy cycles, and time elapsed since completion of RT. All statistical analyses were performed using SSPS 13.0 (SAS Institute, Inc., Cary, North Carolina); *p* values < 0.05 were considered statistically significant.

## Results

### Patient characteristics

A total of 300 patients who had undergone surgery for breast cancer were enrolled in the study; Patients were followed up in March 2017. The valid number of patients is 212 (80 patients were lost to follow-up and eight patients died). The median follow-up time was 27.2 months. The number of patients followed up for 0 to 1.5 years, 1.5 to 2.5 years, 2.5 to 3.5 years and 3.5 to 4.5 years were 64, 61, 66 and 21, respectively. Patient characteristics are listed in Table 1. The mean (±SD) age of patients was 48.36 ± 8.95 years. About 11.3% of patients were illiterate; 28.8% were educated up to primary school level, 43.9% were educated up to middle school and 16.0% of patients were educated up to college level or above. More than 95.3% of patients were married. Most patients had stage II (53.3%) or III (37.3%) disease. Percentages of patients who had received breast-conserving surgery, breast reconstruction surgery and modified radical mastectomy were 22.2%, 5.2% and 72.6%, respectively (Table 1).

**Table 1:** Patients’ characteristics

Variables		Mean ± SD or n (%)
Age (years)		48.36 ± 8.95
Level of education	Illiterate	24 (11.3)
	Primary School	61 (28.8)
	Middle School	93 (43.9)
	College or above	34 (16.0)
Marital status	Unmarried	10 (4.7)
	Married	202 (95.3)
Clinical stage	0	2 (0.9)
	I	11 (5.2)
	II	113 (53.3)
	III	79 (37.3)
	IV	7 (3.3)
Surgical technique	Breast-conserving surgery	47 (22.2)
	Breast reconstruction surgery	11 (5.2)
	Modified radical mastectomy	154 (72.6)

### Univariate analysis of EORTC QLQ-C30 data

Of the 15 subscales, only the role function, social function, pain and financial impact of disease scores exhibited significant changes over time after radiotherapy (*p* < 0.002). Of these, the role function score showed the highest level in the 2.5 to 3.5 years time window post-radiotherapy. Pain scores in the 2.5 to 4.5 years time window post- radiotherapy were significantly lower than those in the 0 to 2.5 years time window, which indicates subsidence of pain over time. The scores for financial impact of disease showed fluctuations over time (45.83 ± 33.60, 42.86 ± 36.67, 18.10 ± 29.53 and 53.33 ± 50.18 in the 0–1.5; 1.5–2.5; 2.5–3.5; and 3.5–4.5 years groups, respectively). The economic impact was found to be lower during the time window of 2.5 to 3.5 years since completion of RT. The total QOL score did not change significantly in different time intervals post RT with respect to the other subscales of function and clinical symptoms (Table 2).

**Table 2:** Results of univariate analysis of EORTC QLQ-C30 questionnaire data

Scale	Time elapsed since completion of radiotherapy				*F*-value	*p*-value
Functional scales	0–1.5 years	1.5–2.5 years	2.5–3.5 years	3.5–4.5 years		
Physical function	72.50 ± 28.17	71.43 ± 22.27	81.71 ± 24.91	90.00 ± 25.38	1.643	0.184
Role function	88.28 ± 17.94	82.86 ± 25.56	95.71 ± 9.56	85.00 ± 24.15	2.715	0.048
Emotional function	89.38 ± 21.54	90.00 ± 13.93	84.00 ± 20.75	81.00 ± 21.83	1.054	0.372
Cognitive function	76.56 ± 22.84	80.00 ± 17.99	71.43 ± 30.40	77.50 ± 14.19	0.788	0.503
Social function	80.73 ± 25.79	79.52 ± 16.71	93.33 ± 16.27	91.67 ± 11.79	4.014	0.009
Symptom scales						
Tiredness	26.82 ± 29.83	23.38 ± 28.43	20.53 ± 27.59	3.99 ± 12.62	1.801	0.151
Nausea/vomiting	2.08 ± 7.02	3.81 ± 17.66	3.33 ± 9.74	0.00 ± 0.00	0.329	0.804
Pain	35.42 ± 31.61	37.14 ± 33.11	18.10 ± 29.53	16.67 ± 23.57	3.261	0.024
Shortness of breath	9.38 ± 29.61	5.71 ± 23.55	11.43 ± 32.28	10.00 ± 31.62	0.238	0.870
Sleep disturbance	9.38 ± 17.42	6.67 ± 17.71	14.29 ± 23.27	10.00 ± 31.62	0.790	0.502
Lack of appetite	9.38 ± 29.61	8.57 ± 28.40	8.57 ± 28.40	10.00 ± 31.62	0.011	0.998
Constipation	7.29 ± 18.42	3.81 ± 10.76	6.67 ± 13.53	20.00 ± 42.16	2.013	0.116
Diarrhea	3.13 ± 17.68	1.43 ± 8.45	1.43 ± 8.45	0.00 ± 0.00	0.243	0.866
Financial impact of disease	45.83 ± 33.60	42.86 ± 36.67	18.10 ± 29.53	53.33 ± 50.18	5.107	0.002
Overall state of health	76.82 ± 14.16	76.67 ± 14.12	68.10 ± 24.63	76.67 ± 10.10	1.863	0.140

### Univariate analysis of EORTC QLQ-BR23 data

Statistically significant between-group differences were observed with respect to scores for perception of own body, sexual activity, enjoyment of sexual activity, concerns about future state of health and symptoms in the breast (*P* < 0.05). The scores for perception of own body increased during the 0 to 3.5 years time window after RT, and then decreased during the 3.5 to 4.5 years time window. Similar trend was observed with respect to the subscales of sexual activity and enjoyment of sexual activity in that the scores dropped significantly during the 2.5 to 4.5 year time window as compared to those during the 0 to 2.5 year time window after completion of RT. On the contrary, the scores for concerns about future state of health rose significantly during the 2.5 to 4.5 year time window as compared with that during the 0 to 2.5 year time window after completion of RT. The scores for subscale of symptoms in the breast fluctuated; the highest scores pertained to the 0 to 1.5 year time window after completion of RT (Table 3).

**Table 3:** Results of univariate analysis of EORTC QLQ-BR23 questionnaire data

Scale	Time elapsed since completion of radiotherapy				*F*-value	*p*-value
Functional scales	0–1.5 years	1.5–2.5 years	2.5–3.5 years	3.5–4.5 years		
Perception of own body	82.29 ± 25.02	90.24 ± 14.50	94.52 ± 11.06	88.33 ± 15.81	2.800	0.043
Sexual activity	81.77 ± 26.22	89.52 ± 22.17	71.90 ± 27.05	76.67 ± 35.31	2.735	0.047
Enjoyment of sexual activity	83.33 ± 26.76	91.43 ± 20.36	71.43 ± 28.17	76.67 ± 35.31	3.546	0.017
Concerns about future state of health	71.88 ± 33.45	74.29 ± 30.61	92.86 ± 17.75	85.00 ± 24.15	4.048	0.009
Symptom scales						
Side-effects of therapy	23.44 ± 23.52	30.00 ± 28.81	21.42 ± 24.11	38.33 ± 23.64	1.549	0.206
Symptoms in the breast	32.81 ± 32.03	17.86 ± 20.63	17.14 ± 22.50	25.00 ± 16.67	2.851	0.041
Symptoms in arm/shoulder	15.66 ± 20.44	13.84 ± 18.00	9.31 ± 9.88	20.05 ± 14.31	1.480	0.224
Low mood because of hair loss	1.56 ± 8.84	4.29 ± 18.67	4.29 ± 14.20	10.00 ± 21.08	0.796	0.499

### Results of multivariate linear regression analysis

On multivariate analysis, time elapsed since completion of RT was an independent predictor of social function, pain, and concerns about future state of health (*p* = 0.003, *p* = 0.011, and *p* = 0.013, respectively) (Tables 4 and 5).

**Table 4:** Variables showing no significant association with time elapsed since radiotherapy on multivariate analyses

Variables	Regression coefficients	Standard error	Standard regression coefficient	*t*	*p*-value
Role function					
Constant term	75.366	18.565			
Age	0.473	0.215	0.214	2.200	0.030
Level of education	2.609	2.232	0.121	1.169	0.245
Surgical technique	−0.220	2.450	−0.009	− 0.090	0.929
Surgical staging	−5.807	3.145	−0.189	−1.846	0.068
Number of chemotherapy cycles	−0.137	0.976	− 0.014	− 0.141	0.888
Time elapsed since completion of RT	2.231	1.977	0.108	1.129	0.262
Financial impact of disease					
Constant term	76.843	31.830			
Age	0.392	0.369	0.095	1.062	0.291
Degree of education	−14.859	3.827	−0.370	−3.883	0.000
Surgical technique	7.793	4.201	0.174	1.855	0.066
Surgical staging	−13.631	5.393	−0.237	−2.528	0.013
Number of chemotherapy cycles	2.638	1.673	0.139	1.576	0.118
Time elapsed since completion of RT	−3.202	3.390	−0.083	−0.944	0.347
Perception of own body					
Constant term	32.595	16.150			
Age	0.429	0.187	0.215	2.292	0.024
Level of education	6.407	1.942	0.329	3.300	0.001
Surgical technique	−0.084	2.131	−0.004	−0.039	0.969
Surgical staging	4.279	2.736	0.154	1.564	0.121
Number of chemotherapy cycles	−0.563	0.849	−0.061	− 0.663	0.508
Time elapsed since `completion of RT	3.219	1.720	0.173	1.871	0.064
Sexual activity					
Constant term	60.288	23.972			
Age	0.810	0.278	0.270	2.916	0.004
Level of education	−4.535	2.882	−0.156	−1.574	0.119
Surgical technique	−0.926	3.164	−0.029	− 0.293	0.770
Surgical staging	3.943	4.061	0.095	0.971	0.334
Number of chemotherapy cycles	−1.596	1.260	−0.116	−1.267	0.208
Time elapsed since completion of RT	−3.547	2.553	−0.127	−1.389	0.168
Enjoyment of sexual activity					
Constant term	55.928	23.971			
Age	0.830	0.278	0.274	2.989	0.003
Level of education	−4.352	2.882	−0.147	−1.510	0.134
Surgical technique	−0.105	3.164	−0.003	− 0.033	0.974
Surgical staging	5.184	4.061	0.123	1.277	0.205
Number of chemotherapy cycles	−1.677	1.260	−0.120	−1.330	0.186
Time elapsed since completion of RT	−4.524	2.553	−0.160	−1.772	0.079
Symptoms in the breast					
Constant term	42.090	24.453			
Age	−0.080	0.283	−0.028	− 0.282	0.778
Level of education	−1.213	2.940	−0.044	−0.413	0.681
Surgical technique	−2.557	3.227	−0.083	−0.792	0.430
Surgical staging	0.934	4.143	0.024	0.225	0.822
Number of chemotherapy cycles	0.097	1.285	0.007	0.076	0.940
Time elapsed since completion of RT	−4.410	2.604	−0.167	−1.693	0.093

**Table 5:** Variables showing a significant association with time elapsed since radiotherapy on multivariate analyses

Variables	Regression coefficients	Standard error	Standard regression coefficient	t-value	*p*-value
Social function					
Constant term	70.104	17.984			
Age	−0.161	0.208	−0.071	−0.770	0.443
Level of education	2.500	2.162	0.114	1.156	0.250
Surgical technique	−5.585	2.373	−0.230	−2.353	0.020
Surgical staging	1.787	3.047	0.057	0.586	0.559
Number of chemotherapy cycles	1.804	0.945	0.175	1.908	0.059
Time elapsed since completion of RT	5.787	1.915	0.276	3.022	0.003
Pain					
Constant term	−0.578	29.509			
Age	0.664	0.342	0.187	1.941	0.055
Level of education	6.030	3.548	0.175	1.700	0.092
Surgical technique	−1.481	3.894	−0.038	−0.380	0.704
Surgical staging	0.919	5.000	0.019	0.184	0.854
Number of chemotherapy cycles	−0.106	1.551	−0.006	− 0.068	0.946
Time elapsed since completion of RT	−8.118	3.143	−0.245	−2.583	0.011
Concerns about future state of health					
Constant term	−0.280	25.906			
Age	0.237	0.300	0.074	0.788	0.432
Level of education	6.092	3.115	0.195	1.956	0.053
Surgical technique	−3.303	3.419	−0.095	−0.966	0.336
Surgical staging	13.289	4.389	0.297	3.028	0.003
Number of chemotherapy cycles	−0.131	1.362	−0.009	− 0.096	0.923
Time elapsed since completion of RT	6.974	2.759	0.233	2.528	0.013

## Discussion

The present study is among the few studies which have evaluated the effect of time elapsed since completion of radiotherapy on the QOL of breast cancer patients following breast surgery using the EORTC QLQ-C30 and EORTC QLQ-BR23 instruments. We found that the patients’ social function, as measured with the EORTC QLQ-C30, improved significantly with passage of time during the 2.5 to 4.5 year time window after completion of RT. Social function refers to the patient’s self-awareness about his/her own health status and the impact on family’s daily life and social activities. A previous study revealed poor QOL of family caregivers of patients with cancers [18]. Strain imposed by cancer associated pain and discomfort, impaired capacity for work, and the dependence on drugs and adjuvant therapy can affect the physical condition of family caregivers, and render them vulnerable to cognitive dysfunction and sleep disorders. The major causes that impair post-treatment social life of patients with breast cancer include physical changes (such as removal of mammary gland), pain, and psychological factors such as depression, irritability and anxiety [19–21]. With passage of time after completion of radiotherapy, patients tend to gradually accept the reality, which helps improve their social adaptability [22–24].

Breast, arm, and shoulder pain are commonly experienced by breast cancer patients after treatment [25, 26]; these were shown to significantly impair the long-term QoL of these patients [27]. These symptoms may be attributable to tumor metastasis, anti-cancer therapy or to social-psychological factors. Previous studies have identified age, axillary lymph node dissection, and postoperative RT as significant risk factors [25, 28]. In the present study, only time elapsed since completion of RT was found to be associated with reduction in pain, after adjusting for age, educational level of patients, operation method, surgical staging and number of chemotherapy cycles; pain relief was especially noticeable after 2.5 to 4.5 years post-radiotherapy.

Scores pertaining to concerns about future state of health showed improvement from 2.5 to 3.5 years post-radiotherapy and a slight decline was also observed during the 3.5 to 4.5 year time window post-radiotherapy. The diagnosis and cure of breast cancer are both typically life-changing events which affect the patient’s psychological health [29]. At initial diagnosis, the patient typically experiences the psychological phenomenon of denial, anger, acceptance, depression, fear and survival, short-term lack of confidence and even fear for future health. Although the negative effects of disease on the patient exist, the positive effects of post-traumatic growth, however, helps augment patient’s confidence and reduces their worries about future [30]. More importantly, the confidence of patients is reinforced by the negative results of the annual cancer re-examination. Additionally, the recovery of functions in various aspects promotes the normal activities of daily living, which enable the patients to look forward to the future [24].

The cross-sectional study design is the main limitation of this study. The design does not allow for causal inferences, but only describes the factors associated with self-reported QoL outcomes.

## Conclusions

In this study cohort, time elapsed since completion of RT had significant impact on social function, pain symptoms, and concerns about future state of health of patients with breast cancer. These scales improved with passage of time after RT. Our results have profound guiding significance for QOL of patients with breast cancer after RT. Of note, breast cancer is a heterogeneous disease and these results are limited to the cross-sectional study. Further studies are needed to elucidate the possible impact of time elapsed since completion of RT on breast cancer patients.

## Abbreviations

EORTC: European Organization Research and Treatment of Cancer
QOL: Quality of life
RT: Radiotherapy
SD: Standard deviation
